# ChatGPT-Assisted Learning Effectiveness and Students’ Academic Outcomes: A Mediation Analysis of Cognitive Engagement and Self-Efficacy

**DOI:** 10.3390/ejihpe16100150

**Published:** 2026-09-27

**Authors:** Ahmed Mohamed Hasanein, Bassam Samir Al-Romeedy, Hazem Ahmed Khairy, Nadir Aliane

**Affiliations:** 1 Management Department, College of Business Administration, King Faisal University, Al-Ahsaa 380, Saudi Arabia; aabdelrazek@kfu.edu.sa (A.M.H.); nhaliane@kfu.edu.sa (N.A.); 2 Tourism Studies Department, Faculty of Tourism and Hotels, University of Sadat City, Sadat City 32897, Egypt; bassam.samir@fth.usc.edu.eg; 3 Hotel Management Department, Faculty of Tourism and Hotels, University of Sadat City, Sadat City 32897, Egypt

**Keywords:** ChatGPT-assisted learning effectiveness, academic performance, cognitive engagement, academic self-efficacy, tourism and hospitality education, higher education

## Abstract

Grounded in Social Cognitive Theory, this study examines the association between ChatGPT-assisted learning effectiveness and students’ academic performance, emphasizing the mediating roles of cognitive engagement and academic self-efficacy. Data were collected from tourism and hospitality students at Egyptian universities. Findings indicate that perceived ChatGPT-assisted learning effectiveness is positively associated with students’ academic performance, cognitive engagement, and academic self-efficacy. Cognitive engagement and academic self-efficacy also show significant positive associations with academic performance, and both constructs significantly mediate the relationship between ChatGPT-assisted learning effectiveness and academic performance. These findings suggest that the educational value students attribute to ChatGPT may operate through complementary cognitive and motivational pathways, while the cross-sectional design prevents causal conclusions.

## 1. Introduction

Tourism and hospitality education has experienced substantial pedagogical transformation in response to rapid digitalization, globalization, and the growing demand for graduates equipped with advanced analytical, problem-solving, and adaptive skills (Firdaus et al., 2025; Khairy & Al-Romeedy, 2024; A. Hasanein & Sobaih, 2023). As a practice-oriented discipline, it requires learning environments that go beyond traditional content delivery, actively fostering student engagement, confidence, and competence in addressing complex, real-world challenges. In this context, educators are increasingly exploring technology-enhanced learning solutions designed to support deeper learning processes and improve academic outcomes.

Generative artificial intelligence (AI), particularly ChatGPT, has emerged as a promising tool capable of delivering interactive feedback, personalized explanations, and immediate academic support (Dos, 2025; Lee et al., 2024; Isiaku et al., 2024). Early studies have explored AI applications in tourism and hospitality education, including instructional trend recognition and AI literacy integration (Zhang et al., 2024). However, the literature remains fragmented, with much research focused on descriptive accounts of technological potential or industry applications rather than theory-driven empirical investigations of learning mechanisms (Lo et al., 2024). In particular, how AI-assisted learning influences cognitive engagement and academic self-efficacy—central determinants of student success—remains underexamined.

Recent perspectives on AI in education increasingly conceptualize generative AI as more than an information-retrieval mechanism. ChatGPT can function as an interactive pedagogical partner by providing immediate explanations, feedback, alternative formulations, and task-oriented support that may scaffold students’ learning processes (Takona, 2024). This perspective is particularly relevant to the present study because it positions ChatGPT as part of the learning environment through which students may organize their cognitive effort and develop confidence while completing academic tasks. Such a view is consistent with the Social Cognitive Theory perspective adopted here, in which environmental resources can shape learners’ cognitive and motivational processes.

The development of generative AI is also moving beyond direct interaction between an individual learner and a single conversational model. Recent research has begun to examine multi-agent LLM environments in which multiple AI agents can assume complementary roles, exchange information, divide complex tasks, and jointly contribute to problem solving (Hang et al., 2026). Related work has further explored the optimization of LLM-driven multi-agent search through heterogeneous groups of agents and reinforcement-learning mechanisms (Chen et al., 2026). These developments suggest that future AI-supported learning environments may involve collaborative configurations in which students interact not with a single AI assistant but with several specialized AI agents performing complementary instructional functions. The present study deliberately focuses on the more established single-agent ChatGPT setting because its empirical model examines students’ perceptions of ChatGPT-assisted learning effectiveness rather than multi-agent collaboration. Nevertheless, the emergence of multi-agent learning environments provides an important direction for extending research on how AI-supported learning affects cognitive engagement, self-efficacy, and academic performance.

The tourism and hospitality education context is theoretically relevant because learning in this field frequently combines conceptual knowledge with applied problem solving, interpersonal scenarios, service operations, and interdisciplinary decision-making. Students may therefore encounter tasks that require them to interpret ambiguous situations, compare alternative solutions, communicate effectively, and transfer classroom knowledge to realistic service contexts. These characteristics may create particular opportunities for conversational AI to function as a learning scaffold by providing explanations, alternative perspectives, simulated scenarios, and iterative feedback. At the same time, the applied nature of hospitality education may increase the risk of inappropriate reliance on AI-generated responses because students may be required to exercise professional judgment rather than simply retrieve factual information. Consequently, the relationships examined in this study should not be interpreted as technology effects that are necessarily identical across academic disciplines. In more technically specialized or laboratory-based fields, for example, learning may depend more heavily on domain-specific experimentation, procedural practice, or physical interaction that cannot be fully reproduced through conversational AI. The tourism and hospitality context therefore provides a theoretically meaningful setting for examining how AI-supported learning interacts with cognitive and motivational learning processes.

Several specific gaps emerge from existing research. First, most studies on ChatGPT in education concentrate on general learning environments or student perceptions, neglecting the psychological and behavioral pathways through which AI affects academic performance (Montenegro-Rueda et al., 2023). Second, although ChatGPT can support understanding, reduce learning-related anxiety, and facilitate task completion (Lee et al., 2024; Ba et al., 2025), empirical work rarely examines the mechanisms linking AI-assisted learning to measurable outcomes, particularly in cognitively demanding contexts such as case analyses, service design, and problem-based learning in tourism and hospitality (Khairy & Al-Romeedy, 2024). Third, learner-centered constructs—such as cognitive engagement and academic self-efficacy—are seldom incorporated into studies of AI-assisted learning, despite their well-established roles in information processing, persistence, and performance (S. Li & Lajoie, 2022; Schunk & DiBenedetto, 2022; Artino, 2012; Pajares, 2012). Cognitive engagement reflects sustained mental effort, higher-order thinking, and self-regulated learning (S. Li & Lajoie, 2022; Barkley & Major, 2020), while academic self-efficacy refers to learners’ beliefs in their ability to successfully complete academic tasks, influencing motivation and persistence (Artino, 2012; Pajares, 2012). Fourth, although Social Cognitive Theory (SCT) offers a robust framework for examining the reciprocal interplay among environmental factors, personal beliefs, and behavioral engagement (Devi et al., 2017; Woreta et al., 2025), it has been underutilized in research on ChatGPT-assisted learning in tourism and hospitality education (Lee et al., 2024; Cai et al., 2024).

To address these gaps, the present study develops and empirically tests a Social Cognitive Theory-based model examining how ChatGPT-assisted learning effectiveness influences academic performance through the mediating effects of cognitive engagement and academic self-efficacy. By integrating these learner-centered constructs, the study investigates the psychological and behavioral pathways that translate AI-supported learning into measurable outcomes.

This research contributes to the literature in three ways. First, it applies a theory-driven framework in an underexamined context characterized by growing adoption of generative AI. Second, it emphasizes learner-centered processes that link technology use to academic outcomes, advancing understanding of how cognitive engagement and self-efficacy mediate the effects of AI-assisted learning. Third, it provides practical guidance for educators aiming to integrate ChatGPT in ways that enhance student engagement, confidence, and academic performance, moving beyond simple technology adoption toward meaningful pedagogical implementation.

## 2. Literature Review and Hypotheses Development

### 2.1. Social Cognitive Theory

Social Cognitive Theory provides a coherent framework for understanding how ChatGPT-assisted learning effectiveness influences students’ academic performance through key psychological and behavioral mechanisms. The theory conceptualizes learning as the result of reciprocal interactions between environmental conditions, personal beliefs, and learners’ behaviors, emphasizing that academic outcomes emerge from these interrelated processes rather than from technological exposure alone (Devi et al., 2017; Woreta et al., 2025). From this perspective, ChatGPT-assisted learning represents an environmental factor that can shape students’ learning experiences by offering timely feedback, adaptive explanations, and a supportive space for academic interaction (Isiaku et al., 2024). Such characteristics can alter students’ perceptions of academic tasks, reducing perceived difficulty and enhancing their sense of control over learning activities (Lee et al., 2024). As a result, learners are more likely to approach academic challenges with confidence rather than avoidance (Cai et al., 2024).

Within Social Cognitive Theory, academic self-efficacy plays a central role in translating environmental support into meaningful learning behaviors (Woreta et al., 2025; Al-Romeedy et al., 2020). When students perceive themselves as capable of understanding and managing academic tasks, they demonstrate greater persistence, confidence, and willingness to invest effort (Conley & French, 2014). Effective engagement with ChatGPT can strengthen these beliefs by enabling successful task completion and reinforcing students’ trust in their own academic abilities (Lee et al., 2024). These strengthened self-beliefs are closely associated with higher levels of cognitive engagement, as students who believe in their competence are more inclined to engage deeply with learning materials, apply critical thinking strategies, and sustain attention during cognitively demanding tasks (Bobis et al., 2016; Pohl, 2020). Cognitive engagement thus reflects the behavioral dimension through which personal beliefs influence academic learning processes (S. Li & Lajoie, 2022).

In this way, Social Cognitive Theory explains how ChatGPT-assisted learning effectiveness contributes to academic performance by shaping students’ self-efficacy and cognitive engagement as interconnected but non-sequential mechanisms. This theoretical perspective supports the proposed model by highlighting the indirect pathways through which intelligent learning environments influence academic outcomes.

The selection of cognitive engagement and academic self-efficacy reflects two complementary dimensions of the learning process emphasized by Social Cognitive Theory. Cognitive engagement captures the extent to which students invest mental effort and actively process learning content, whereas academic self-efficacy captures their beliefs about their capability to manage academic demands. These constructs therefore represent distinct but complementary mechanisms through which an external learning environment may be associated with academic outcomes. Treating them as parallel mediators is theoretically appropriate because ChatGPT may simultaneously provide cognitive support that facilitates active processing and psychological support that strengthens perceived capability. The model consequently moves beyond a simple technology-to-performance relationship by examining whether perceived effectiveness of ChatGPT is associated with academic performance through both a cognitive pathway and a motivational pathway.

### 2.2. ChatGPT-Assisted Learning Effectiveness and Academic Performance

Learning effectiveness in technology-supported environments extends beyond the mere presence of digital tools to encompass the extent to which such tools actively shape learners’ academic experiences and performance (A. Hasanein & Sobaih, 2023). When ChatGPT-assisted learning operates effectively, it restructures the learning environment by offering timely guidance, adaptive explanations, and continuous academic support that directly influence how students approach and execute academic tasks (Lee et al., 2024). These features reduce ambiguity, minimize trial-and-error learning, and allow students to focus their cognitive resources on task completion rather than on navigating instructional uncertainty (Watson et al., 2024). From a social cognitive standpoint, environments that consistently facilitate successful task execution enhance learners’ capacity to perform academically by creating conditions that support mastery and reduce performance-related constraints (Huawei & Jenatabadi, 2024). In such contexts, academic performance is not only the result of individual effort but also a reflection of how effectively the learning environment enables students to translate effort into outcomes (Khairy & Al-Romeedy, 2024). When students interact with an effective ChatGPT-assisted learning system, they are more likely to complete assignments accurately, manage academic requirements efficiently, and demonstrate higher levels of academic achievement, as the system supports the behavioral enactment of learning in a structured and responsive manner (Al-Abri, 2025; A. Hasanein et al., 2024). Accordingly, the present study proposes the following hypothesis:

**H1.**  *ChatGPT-assisted learning effectiveness is positively associated with students’ academic performance.*

### 2.3. ChatGPT-Assisted Learning Effectiveness and Cognitive Engagement

The extent to which students engage cognitively with learning tasks is largely shaped by how stimulating and responsive the instructional environment is (Akdeniz, 2024). Environments that merely transmit information tend to elicit surface-level processing, whereas those that invite interaction, inquiry, and reflection encourage deeper mental involvement (Al Mamun & Lawrie, 2024). When ChatGPT-assisted learning is perceived as effective, it introduces an interactive dimension that shifts students from passive recipients of information to active participants in the learning process (Ba et al., 2025). Rather than simply consuming content, students are encouraged to question, elaborate, and refine their understanding through continuous dialogue (Zwiers & Crawford, 2023). Within socially constructed learning contexts, such interactive conditions play a crucial role in directing learners’ cognitive investment (Kharroubi & ElMediouni, 2024). An effective ChatGPT system supports sustained attention by breaking down complex ideas, responding to individual learning needs, and maintaining an ongoing exchange that keeps students mentally engaged with academic material (A. Hasanein et al., 2024). As a result, students are more likely to apply higher-order thinking strategies, persist in cognitively demanding activities, and approach learning tasks with intentional focus (Casagrand & Semsar, 2017). Accordingly, the following hypothesis is proposed:

**H2.**  *ChatGPT-assisted learning effectiveness is positively associated with students’ cognitive engagement.*

### 2.4. ChatGPT-Assisted Learning Effectiveness and Academic Self-Efficacy

Students’ beliefs about their academic capabilities are not formed in isolation but are continuously shaped by the learning conditions in which they operate (Alam & Mohanty, 2023). When instructional environments provide consistent guidance, constructive feedback, and opportunities for successful task completion, they contribute to the development of stronger academic self-efficacy beliefs (Brown et al., 2016). Effective ChatGPT-assisted learning offers such conditions by enabling students to clarify misunderstandings, test their knowledge, and receive immediate, non-evaluative responses that reduce anxiety associated with academic failure (Lee et al., 2024; A. Hasanein & Sobaih, 2023). Through repeated interaction with an effective ChatGPT system, students are more likely to experience a sense of mastery over academic tasks, as challenges become manageable and progress becomes visible (Isiaku et al., 2024). These mastery-oriented experiences reinforce students’ confidence in their ability to understand course material, solve problems, and meet academic demands (Rhodes, 2022). As confidence in one’s academic competence strengthens, students are better equipped to approach learning tasks with assurance rather than doubt (A. M. Hasanein, 2025). Consequently, the following hypothesis is suggested:

**H3.**  *ChatGPT-assisted learning effectiveness is positively associated with students’ academic self-efficacy.*

An important conceptual distinction should nevertheless be recognized. Academic self-efficacy refers to students’ beliefs about their capability to successfully perform academic tasks, whereas confidence that emerges specifically when AI assistance is available may represent a more context-dependent form of task confidence. In the present study, the measurement items explicitly situate students’ confidence within ChatGPT-assisted learning. Accordingly, the hypothesized relationship should be interpreted as an association between perceived ChatGPT-assisted learning effectiveness and academic self-efficacy as expressed in an AI-supported learning context, rather than as definitive evidence that ChatGPT strengthens students’ independent academic capabilities. This distinction is consistent with the reciprocal logic of Social Cognitive Theory while highlighting the need to examine whether efficacy beliefs remain stable when AI assistance is unavailable.

### 2.5. Cognitive Engagement and Students’ Academic Performance

Academic performance reflects not only what students know, but also how effectively they process, interpret, and apply academic knowledge across learning situations (Khairy & Al-Romeedy, 2024). Cognitive engagement represents this qualitative dimension of learning, as it captures students’ willingness to invest sustained mental effort, actively regulate their understanding, and employ purposeful strategies when dealing with academic tasks (S. Li & Lajoie, 2022). Students who are cognitively engaged tend to approach learning activities with intention, continuously evaluating their comprehension and adjusting their efforts to meet task demands (Barkley & Major, 2020). Such active mental involvement plays a critical role in translating instructional input into measurable academic outcomes (Gettinger & Walter, 2012). When students remain cognitively engaged, they are more capable of handling complex material, maintaining focus during challenging tasks, and persisting despite academic difficulties (Skinner et al., 2016). This depth of processing allows learners to move beyond surface-level memorization toward meaningful understanding, which is more likely to be reflected in higher academic achievement (Khairy & Al-Romeedy, 2024). So, the following hypothesis is developed:

**H4.**  *Cognitive engagement is positively associated with students’ academic performance.*

### 2.6. Academic Self-Efficacy and Students’ Academic Performance

Students’ academic performance is strongly influenced by how they perceive their own ability to successfully manage academic demands (Mulaudzi, 2023). Academic self-efficacy shapes not only students’ expectations of success but also the intensity of effort they are willing to invest and their persistence when confronted with academic challenges (Schunk & DiBenedetto, 2022). Students who believe in their academic competence are more likely to approach tasks proactively, maintain focus under pressure, and recover more effectively from setbacks, all of which contribute to stronger academic outcomes. These beliefs play a critical role in determining how students translate learning opportunities into performance (Kao, 2024; X. Li, 2018). High academic self-efficacy encourages learners to set challenging goals, engage more confidently with complex material, and sustain effort over time, even in demanding academic contexts (Schunk & DiBenedetto, 2022). As a result, students with stronger self-efficacy beliefs are better positioned to perform consistently and effectively across a range of academic tasks (Artino, 2012). Hence, the following hypothesis is assumed:

**H5.**  *Academic self-efficacy is positively associated with students’ academic performance.*

### 2.7. The Mediating Role of Cognitive Engagement

The influence of ChatGPT-assisted learning effectiveness on academic performance is unlikely to occur solely through direct exposure to technological support, but rather through the learning behaviors that such support activates (Ba et al., 2025; Cai et al., 2024). While an effective ChatGPT system can enhance access to information and instructional clarity, its impact on academic outcomes ultimately depends on how students cognitively engage with the learning process (A. M. Hasanein, 2025). Cognitive engagement represents the mechanism through which environmental learning support is transformed into meaningful academic action, as it captures the extent to which students invest mental effort, sustain focus, and actively process academic content (Alam & Mohanty, 2024; Khairy & Al-Romeedy, 2024). When ChatGPT-assisted learning is perceived as effective, it creates conditions that encourage students to interact more deeply with academic material, prompting reflection, strategic thinking, and sustained involvement in learning tasks (Lee et al., 2024). This heightened cognitive engagement enables students to process information more thoroughly, apply knowledge more effectively, and persist through challenging academic activities, thereby contributing to improved academic performance (A. Hasanein et al., 2024). In this sense, cognitive engagement serves as a critical explanatory pathway that links the quality of the learning environment to students’ academic outcomes (Guo et al., 2022).

The proposed mediating logic is also consistent with broader evidence from digital learning research showing that technological and environmental conditions do not necessarily translate directly into performance outcomes but may operate through learners’ internal cognitive and self-regulatory processes. Essuman et al. (2025), for example, demonstrate the relevance of digital literacy, motivation, self-regulation, interest, and task-related conditions in explaining performance within online learning environments. Although their model differs from the present ChatGPT-focused framework, the findings support the broader proposition that technology-related learning conditions may become educationally meaningful through internal learner processes. The present study extends this reasoning by examining cognitive engagement and academic self-efficacy as complementary pathways connecting perceived ChatGPT-assisted learning effectiveness with academic performance. Accordingly, this study proposes the following hypothesis:

**H6.**  *Cognitive engagement mediates the association between ChatGPT-assisted learning effectiveness and students’ academic performance*

### 2.8. The Mediating Role of Academic Self-Efficacy

Academic self-efficacy represents a central psychological resource through which students interpret and respond to learning support provided by digital environments (Pan, 2022). Rather than influencing academic performance directly, effective instructional technologies shape performance by altering how capable students feel when engaging with academic tasks (Khairy & Al-Romeedy, 2024). In the context of ChatGPT-assisted learning, effectiveness is reflected in the system’s ability to help students resolve confusion, structure their learning efforts, and navigate academic challenges with greater confidence (Lee et al., 2024; Ba et al., 2025). As students repeatedly experience successful task completion and clearer understanding through interaction with an effective ChatGPT system, their beliefs about their own academic competence are gradually reinforced (Rasul et al., 2023). These strengthened beliefs influence how students approach academic tasks, affecting their persistence, goal commitment, and willingness to confront difficulty (Pajares, 2012). Consequently, improvements in academic performance can be understood as the outcome of enhanced self-efficacy rather than as a direct consequence of technological support alone (A. M. Hasanein, 2025). Based on this reasoning, the following hypothesis is formulated:

**H7.**  *Academic self-efficacy mediates the association between ChatGPT-assisted learning effectiveness and students’ academic performance.*

To elucidate the hypothesized relationships and underlying mechanisms, the conceptual framework of this study is presented in Figure 1. The figure illustrates how ChatGPT-assisted learning impacts hospitality and tourism students’ academic performance both directly and indirectly, operating through two parallel mediating pathways involving cognitive engagement and academic self-efficacy.

## 3. Method

### 3.1. Sampling and Data Collection

This study investigates the relationship between ChatGPT-assisted learning effectiveness (CALE) and academic performance (AP) among hospitality and tourism students, with particular attention to the mediating roles of cognitive engagement (CE) and academic self-efficacy (ASE). The conceptual model was established based on a comprehensive examination of relevant theoretical and empirical research, which provided the basis for developing the proposed hypotheses and specifying the relationships among the study constructs. To empirically examine the proposed model, primary data were obtained through a structured questionnaire administered to hospitality and tourism students enrolled at public universities in Egypt. The collected data were analyzed using Partial Least Squares Structural Equation Modeling (PLS-SEM), which enabled simultaneous evaluation of the measurement model and structural relationships, including the proposed indirect relationships. In addition, procedural and statistical approaches were incorporated to assess the possibility of common method bias (CMB).

Before launching the main survey, a pilot investigation was undertaken with 40 hospitality and tourism students who had previously used ChatGPT for academic purposes. The purpose of the pilot was to determine whether the questionnaire items were clear, understandable, and suitable for the intended educational setting, consistent with the recommendations of Presser et al. (2004) and Dillman et al. (2014). Participants provided feedback through brief interviews and written observations, with particular emphasis on item wording, response direction, and contextual relevance. The resulting feedback led to minor wording modifications intended to improve readability, remove potential ambiguity, and ensure that the instrument appropriately reflected the educational context under investigation.

A purposive sampling technique was employed because participation was subject to predefined eligibility requirements. Specifically, respondents were required to be hospitality or tourism students enrolled at an Egyptian public university and to have previous experience using ChatGPT in academic learning activities. Consequently, the sampling frame was restricted to students satisfying these criteria rather than the broader population of university students. Potential participants were reached through academic contacts, student organizations, university course groups, peer referrals, and relevant social-media channels. Data collection took place across nine Egyptian public universities from October to December 2025. Given the non-probability nature of the purposive sampling procedure, the findings should be understood as evidence concerning the participating student sample and should not be interpreted as statistically representative of all university students in Egypt.

The questionnaire was provided in both Arabic and English to ensure linguistic suitability and facilitate respondents’ comprehension. The equivalence of the two language versions was examined through a back-translation procedure conducted by two bilingual experts. In addition, a preliminary assessment involving 30 hospitality and tourism faculty members was conducted to evaluate the clarity, organization, and comprehensibility of the questionnaire. Minor modifications were subsequently introduced in response to their observations to improve readability and contextual appropriateness.

The cultural, linguistic, and institutional characteristics of Egyptian higher education are also relevant to the interpretation of the study findings. The availability of the questionnaire in both Arabic and English allowed respondents to select the language they found more suitable or comfortable; participants were not assigned to separate Arabic- and English-language groups. Nevertheless, variations in English-language proficiency and familiarity with academic terminology may affect how some students understand concepts related to generative artificial intelligence, particularly given the substantial presence of English in technical AI discourse and AI interfaces. Moreover, students’ perceptions and patterns of ChatGPT use may be influenced by features of the Egyptian public-university environment, including established expectations regarding instructor authority, conventional assessment practices, familiarity with digital technologies, and differing levels of exposure to generative AI. Students who primarily view instructors as authoritative academic guides, for instance, may regard ChatGPT as a complementary source of explanation and support rather than as an alternative to instructor guidance. Likewise, differences in prior experience with generative AI may shape whether ChatGPT is employed for exploration and explanation, checking and verification, or generating relatively complete responses.

Such contextual characteristics may have implications for students’ cognitive engagement and their perceptions of their academic capabilities. Accordingly, the Egyptian setting represents more than a geographically underexamined research context. It constitutes a particular emerging-economy educational environment in which linguistic practices, institutional expectations, technological familiarity, and assessment norms may shape students’ experiences with AI-supported learning. The findings should therefore be interpreted within these contextual conditions and should not be assumed to apply uniformly to higher-education systems characterized by different cultural, linguistic, technological, and pedagogical environments.

Several procedural and statistical strategies were implemented to address the potential for common method bias in accordance with Podsakoff et al. (2003). At the procedural level, respondents were informed that their participation was anonymous and that the information collected would be used exclusively for research purposes. Questionnaire items were formulated to avoid ambiguous or leading wording, while predictor and criterion constructs were presented in separate sections to reduce the likelihood of respondents attempting to maintain consistency across related responses. At the statistical level, Harman’s single-factor procedure was initially employed as a diagnostic, and the largest unrotated factor explained 34% of the total variance. In addition, full-collinearity variance inflation factors (VIFs) were examined as a further assessment of potential common method bias. All full-collinearity VIF values were below the conservative threshold of 3.3 proposed by Kock (2015). Collectively, these procedural and statistical assessments indicate that CMB is unlikely to constitute a substantial threat to the findings, although residual method effects cannot be completely excluded because the study uses self-reported data collected at a single point in time.

### 3.2. Measures

ChatGPT-assisted learning effectiveness (CALE) was measured using five items adapted from Cai et al. (2024). Cognitive engagement (CE) was assessed with seven items derived from the measures reported by Miller et al. (1996) and Dınh (2023). These items were adapted to capture students’ cognitive involvement in academic learning activities supported by ChatGPT while maintaining the conceptual meaning of the original measures. Academic self-efficacy (ASE) was measured using five items adapted from Brown et al. (2016). Academic performance (AP) was assessed through three items adopted from Duong et al. (2025), which were designed to evaluate academic performance among university students. All adapted measures were examined during the expert-review and pilot stages to ensure their clarity, contextual suitability, and consistency with the conceptual definitions of the respective constructs. The complete measurement items and their sources are reported in Appendix A.

Content validity was established through an expert assessment involving academics with relevant expertise in hospitality education, educational technology, and learning psychology. The experts evaluated the measurement items in terms of clarity, contextual appropriateness, and consistency with the conceptual framework. Their recommendations resulted in minor revisions aimed at improving the precision of item wording, conceptual clarity, and cultural appropriateness. The internal consistency of the measurement scales was subsequently assessed using Cronbach’s alpha (α). All constructs were operationalized using a seven-point Likert response format ranging from 1 = strongly disagree to 7 = strongly agree.

### 3.3. Data Analysis

In total, 700 questionnaires were distributed among hospitality and tourism students enrolled at public universities in Egypt who had experience using ChatGPT for educational purposes. A total of 574 questionnaires were returned in complete and usable form, representing an 82% response rate, with no missing observations. The final sample comprised 329 males (57.3%) and 245 females (42.7%). Regarding age, 56.7% of the participants were between 20 and 25 years old, while 59.4% were junior or senior students. This distribution reflects the substantial representation of upper-level undergraduate students within hospitality and tourism education.

Although the initial assessment considered the conventional recommendation of at least 10 respondents per survey item, in line with Nunnally and Bernstein (1994), a more rigorous a priori power analysis was subsequently conducted to establish the adequacy of the final sample. The analysis was performed using G*Power version 3.1.9.7 and was based on the endogenous construct with the greatest number of predictors in the structural model, namely Academic Performance (AP). AP is directly predicted by ChatGPT-Assisted Learning Effectiveness (CALE), Cognitive Engagement (CE), and Academic Self-Efficacy (ASE), resulting in three predictors. Using an F-test for linear multiple regression (fixed model, R^2^ deviation from zero), a conservative small effect size of f^2^ = 0.02, an alpha level of 0.05, and statistical power of 0.80, the analysis indicated a minimum required sample size of 550 participants. The final sample of 574 students therefore exceeded the minimum required number, providing adequate statistical power for detecting small effects within the proposed structural model.

The data analysis process was conducted in two main stages. First, SPSS version 26 was used for preliminary data screening and descriptive analysis to verify the completeness and accuracy of the dataset. Second, SmartPLS version 4 was employed to evaluate the measurement and structural models following the two-stage SEM procedure described by Leguina (2015). PLS-SEM was selected because of its appropriateness for predictive and exploratory studies, particularly in behavioral and educational research where multivariate normality may not necessarily be satisfied (Henseler et al., 2009; Hair et al., 2017; Do Valle & Assaker, 2016).

The measurement model was assessed before examining the structural relationships. Indicator reliability was evaluated through the standardized outer loadings and their statistical significance, while construct reliability was examined using Cronbach’s alpha and composite reliability. Convergent validity was assessed through the average variance extracted (AVE), and discriminant validity was examined using the Fornell–Larcker criterion and the heterotrait–monotrait (HTMT) ratio. The measurement-model assessment was conducted using the four constructs specified in the conceptual framework: CALE, CE, ASE, and AP.

The structural model was subsequently evaluated by examining the estimated path coefficients, their significance levels, and the associated effect sizes. Bootstrapping was used to assess the statistical significance of the hypothesized relationships and indirect effects. In addition, predictive relevance was assessed using the appropriate PLS-SEM predictive-relevance procedure. These analyses provided the basis for evaluating the proposed direct and parallel mediating relationships.

Potential common method variance was also assessed statistically in addition to the procedural safeguards described in Section 3.1. Following Podsakoff et al. (2003), Harman’s single-factor procedure was used as an initial diagnostic and indicated that no single factor accounted for the majority of the observed variance. Furthermore, the full-collinearity VIF procedure was applied to identify excessive collinearity that could signal common method bias. All full-collinearity VIF values were below 3.3, consistent with the conservative criterion suggested by Kock (2015). The combined evidence from the procedural precautions and statistical diagnostics indicates that common method bias is unlikely to materially distort the reported relationships, while recognizing that the use of cross-sectional self-reported data means that method-related influences cannot be entirely ruled out.

## 4. Results

### Measurement Model

To assess the potential presence of common method variance (CMV), an Exploratory Factor Analysis (EFA) was performed on the 20 measurement items. The results indicated that the largest unrotated factor explained 34% of the total variance, which is substantially below the 50% benchmark recommended by Podsakoff et al. (2003). This finding suggests that CMV is unlikely to pose a significant threat to the validity of the study’s findings. Convergent validity and internal consistency were evaluated using Average Variance Extracted (AVE), Composite Reliability (CR), and Cronbach’s alpha coefficients. In accordance with the criteria proposed by Hair et al. (2017) and Fornell and Larcker (1981), AVE values equal to or exceeding 0.50 indicate that a construct captures at least half of the variance of its indicators, CR values above 0.70 signify satisfactory internal consistency, and Cronbach’s alpha values of 0.70 or higher reflect acceptable reliability (Nunnally & Bernstein, 1994). Furthermore, standardized outer loadings greater than 0.70 demonstrate that individual indicators contribute meaningfully to their corresponding constructs (Hair et al., 2017).

All constructs satisfied these recommended thresholds, confirming that each latent variable explained more than 50% of the variance in its indicators and demonstrated strong internal consistency. As shown in Table 1, all item loadings exceeded 0.70, indicating a high level of indicator reliability. In addition, Variance Inflation Factor (VIF) values for all indicators were below the critical value of 5, suggesting the absence of multicollinearity concerns (Hair et al., 2017; Kock, 2015). Collectively, these results indicate that the measurement model exhibits sound psychometric properties, providing robust evidence of reliability and validity and supporting the suitability of the constructs for subsequent structural model evaluation (Hair et al., 2017; Henseler et al., 2009; Fornell & Larcker, 1981).

The discriminant validity of the measurement model was assessed using both the Fornell–Larcker criterion and the heterotrait–monotrait ratio (HTMT). As reported in Table 2, the square root of the AVE for each construct exceeds its correlations with the other constructs, satisfying the Fornell–Larcker criterion. In addition, all HTMT values remain below the recommended threshold of 0.90, indicating adequate empirical distinctiveness among ChatGPT-Assisted Learning Effectiveness, Cognitive Engagement, Academic Self-Efficacy, and Academic Performance. These results provide evidence that the constructs capture conceptually distinguishable dimensions of the proposed model.

As presented in Table 3 and Figure 2, all hypothesized relationships in the proposed model were positive and statistically significant, providing empirical support for the proposed research framework. The results indicate positive associations between ChatGPT-assisted learning effectiveness and students’ academic performance, cognitive engagement, and academic self-efficacy. With respect to the direct associations, H1 examined the relationship between ChatGPT-assisted learning effectiveness and academic performance. The findings indicate a statistically significant positive association (β = 0.293, T = 2.880, *p* = 0.004), suggesting that students who perceive ChatGPT as an effective learning support tend to report higher levels of academic performance. This finding is consistent with the proposed relationship between AI-assisted learning and students’ academic outcomes.

H2 examined the association between ChatGPT-assisted learning effectiveness and cognitive engagement and was statistically supported (β = 0.681, T = 6.152, *p* < 0.001). This finding indicates that higher perceived effectiveness of ChatGPT-assisted learning is associated with higher levels of cognitive engagement in learning activities, including sustained attention, deep processing, and active participation. Similarly, H3 examined the association between ChatGPT-assisted learning effectiveness and academic self-efficacy and revealed a statistically significant positive association (β = 0.736, T = 5.839, *p* < 0.001). This indicates that students reporting greater perceived effectiveness of ChatGPT-assisted learning also tend to report stronger academic self-efficacy. Furthermore, H4 examined the association between cognitive engagement and academic performance and was supported (β = 0.353, T = 4.508, *p* < 0.001), indicating that higher cognitive engagement is associated with higher reported academic performance. H5 was also supported, with academic self-efficacy showing a statistically significant positive association with academic performance (β = 0.302, T = 3.075, *p* = 0.002).

Regarding the indirect associations, H6 examined whether cognitive engagement statistically mediates the association between ChatGPT-assisted learning effectiveness and academic performance. The results revealed a significant indirect association (β = 0.240, T = 4.409, *p* < 0.001). Similarly, H7 examined the mediating role of academic self-efficacy and revealed a significant indirect association (β = 0.223, T = 3.117, *p* = 0.002). These findings indicate that the positive association between perceived ChatGPT-assisted learning effectiveness and reported academic performance is statistically transmitted through cognitive engagement and academic self-efficacy. Collectively, the findings provide empirical support for the proposed direct and parallel indirect associations, while the cross-sectional design means that these statistical relationships should not be interpreted as evidence of causal effects.

## 5. Discussion

This study investigated the role of ChatGPT-assisted learning in shaping academic performance among tourism and hospitality higher education students in Egyptian universities, with particular emphasis on the mediating mechanisms of cognitive engagement and academic self-efficacy. Anchored in Social Cognitive Theory, the findings collectively suggest that perceived ChatGPT-assisted learning effectiveness is associated with cognitive and motivational processes relevant to academic performance.

The observed positive relationship between ChatGPT-assisted learning effectiveness and academic performance is consistent with a growing body of empirical research highlighting the instructional value of generative AI systems.

Prior studies suggest that AI-driven tools may support academic performance by providing immediate feedback, clarifying complex concepts, and supporting task completion in a responsive learning environment (Al-Abri, 2025; A. Hasanein et al., 2024). The present findings extend this evidence by demonstrating that, in tourism and hospitality education, higher perceived effectiveness of ChatGPT-assisted learning is associated with more favorable reported academic outcomes. This aligns with Elhag and Yousef (2025), who argue that structured integration of ChatGPT can improve comprehension and sustain learning momentum. From a Social Cognitive Theory perspective, these results reinforce the idea that academic performance emerges from the interaction between learners’ efforts and enabling environmental conditions, rather than from individual effort alone (Khairy & Al-Romeedy, 2024).

Beyond its direct association with academic performance, ChatGPT-assisted learning effectiveness was positively associated with cognitive engagement. This finding supports earlier research indicating that AI-supported learning environments promote sustained attention and deeper mental involvement by adapting explanations to learners’ needs and maintaining interactive exchanges (A. Hasanein et al., 2024). Such engagement encourages students to employ higher-order cognitive strategies, persist through intellectually demanding tasks, and approach learning with deliberate focus, as suggested by Casagrand and Semsar (2017). Moreover, the systematic review by Lo et al. (2024) provides convergent evidence that embedding ChatGPT within instructional activities increases situational engagement and active learning behaviors. The current findings are consistent with the view that generative AI may be associated with cognitive engagement, a core mechanism through which academic success is achieved in Social Cognitive Theory.

Nevertheless, the positive association observed in this study should not be interpreted as evidence that greater engagement with ChatGPT necessarily produces deeper learning. Conversational interaction with an AI system may increase students’ attention and activity while simultaneously encouraging over-reliance on externally generated explanations or answers. Students may, for example, accept plausible AI-generated information without independently checking its accuracy, substitute generated responses for their own reasoning, or develop confidence that is disproportionate to their actual understanding. Such possibilities are particularly important because the effectiveness of AI-assisted learning depends not only on the availability of cognitive support but also on how students use that support. The present findings therefore indicate an association between perceived ChatGPT-assisted learning effectiveness and cognitive engagement, rather than establishing that ChatGPT use invariably produces higher-quality or deeper learning. Future research should distinguish productive AI-supported engagement from dependency-oriented use and examine whether the educational consequences of ChatGPT vary according to the extent to which students independently verify, critique, and reconstruct AI-generated content.

The positive association between ChatGPT-assisted learning effectiveness and academic self-efficacy further highlights the motivational influence of AI-supported learning. Consistent with prior studies, interaction with generative AI is positively associated with students’ confidence in their academic abilities by offering task scaffolding and constructive feedback, which are known to foster mastery experiences (Wu et al., 2025). These results align with established research emphasizing self-efficacy as a critical determinant of motivation and performance, particularly when learners perceive themselves as capable of meeting academic demands (Rhodes, 2022). As A. M. Hasanein (2025) notes, such confidence enables students to approach learning tasks with assurance, reducing anxiety and increasing persistence. Thus, perceived ChatGPT-assisted learning effectiveness is associated not only with students’ perceptions of information support but also with their beliefs about their academic competence. The positive association between ChatGPT-assisted learning effectiveness and academic self-efficacy also requires careful interpretation. The findings may reflect genuine efficacy-building experiences when students use ChatGPT to clarify difficult concepts, obtain feedback, and successfully overcome academic challenges. However, because the present measurement captures confidence within a ChatGPT-assisted learning context, the observed relationship may also reflect confidence in completing academic tasks when external AI support is available. This distinction is important because confidence supported by an external tool does not necessarily indicate stronger independent capability. Future studies should therefore compare students’ academic self-efficacy under AI-assisted and non-AI conditions and examine whether perceived mastery persists after ChatGPT support is removed.

The findings also confirm that both cognitive engagement and academic self-efficacy exert independent positive effects on academic performance. This pattern is well documented in educational psychology literature, which consistently links deeper engagement and stronger self-beliefs to improved academic outcomes. Self-efficacious students are more inclined to set challenging goals, invest sustained effort, and remain resilient in the face of difficulty (Schunk & DiBenedetto, 2022). Similarly, cognitively engaged learners are more likely to engage in meaningful processing rather than surface-level memorization, resulting in higher achievement (Khairy & Al-Romeedy, 2024). These relationships reinforce Social Cognitive Theory’s emphasis on internal cognitive and motivational processes as key drivers of performance.

Crucially, the mediation analysis indicates that cognitive engagement and academic self-efficacy statistically account for part of the association between perceived ChatGPT-assisted learning effectiveness and academic performance. Rather than exerting a purely direct effect, the observed associations are consistent with the possibility that cognitive and motivational processes are relevant to the relationship between perceived ChatGPT-assisted learning effectiveness and academic performance. This finding aligns with A. M. Hasanein (2025), who emphasizes that technological tools yield academic benefits only when they stimulate active cognitive involvement. The mediating roles identified in this study underscore the importance of viewing AI-assisted learning as a process-oriented intervention that enhances learning through psychological mechanisms rather than as a standalone solution.

In practical terms, ChatGPT-assisted learning effectiveness is reflected in its capacity to help students resolve misunderstandings, organize learning activities, and navigate academic challenges with greater confidence (Lee et al., 2024; Ba et al., 2025). The positive association with academic performance can therefore be interpreted in relation to students’ reported self-efficacy and cognitive engagement, rather than as evidence of a direct technological effect or as direct consequences of technological access alone (A. M. Hasanein, 2025). This distinction is particularly relevant in tourism and hospitality education, where applied learning and problem-solving require both cognitive investment and confidence in one’s abilities.

Another consideration concerns the heterogeneity of ChatGPT-supported learning behaviors. The present study treats ChatGPT-assisted learning effectiveness as a general construct and therefore does not distinguish among different purposes of use. Yet the educational meaning of ChatGPT interaction may differ substantially across activities. Using the system to challenge an argument, identify weaknesses in a proposed solution, generate alternative explanations, or provide feedback may encourage active knowledge construction. In contrast, asking ChatGPT to generate a complete assignment or answer may reduce the amount of independent cognitive work performed by the student. Consequently, two students may report similarly high levels of perceived ChatGPT-assisted learning effectiveness while engaging in fundamentally different learning behaviors. The positive relationships observed in the present study should therefore be interpreted at the construct level and should not be taken to mean that every form of ChatGPT use has equivalent educational value.

The Egyptian educational context may also shape how students interpret and use ChatGPT. The questionnaire was administered in Arabic and English, and bilingual translation and back-translation procedures were used to support linguistic equivalence. Nevertheless, students’ language proficiency may influence how they formulate prompts, evaluate AI-generated explanations, and judge the usefulness of ChatGPT responses. In addition, educational experiences characterized by relatively strong instructor authority may influence students’ expectations concerning the role of teachers versus technology in learning. Differences in technology familiarity, prior exposure to generative AI, digital literacy, and assessment practices may likewise affect students’ perceptions of AI-supported learning. These contextual characteristics suggest that the positive relationships identified in the Egyptian sample should be interpreted as evidence from a specific educational and cultural environment rather than as universally invariant relationships. Cross-national studies could therefore examine whether language, educational norms, AI familiarity, and institutional assessment practices moderate the relationships among ChatGPT-assisted learning, self-efficacy, cognitive engagement, and academic performance.

## 6. Theoretical Implications

This study offers several important theoretical contributions to the growing body of literature on technology-enhanced learning by extending the application of Social Cognitive Theory (SCT) within the context of artificial intelligence-assisted education. Specifically, it advances understanding of how ChatGPT-assisted learning effectiveness is associated with students’ academic outcomes through key cognitive and motivational mechanisms among tourism and hospitality higher education students in Egypt.

First, the findings reinforce the relevance of Social Cognitive Theory in explaining learning processes in AI-supported educational environments. SCT posits that learning outcomes result from reciprocal interactions among environmental factors, cognitive processes, and behavioral outcomes. By conceptualizing ChatGPT-assisted learning effectiveness as a salient environmental factor, this study provides empirical evidence of positive associations between perceived AI-assisted learning effectiveness, students’ cognitive engagement and academic self-efficacy, and reported academic performance. This extends SCT beyond traditional instructional settings and validates its applicability in emerging AI-mediated learning contexts.

Second, the study contributes to theory by empirically identifying cognitive engagement as a critical explanatory mechanism linking AI-assisted learning to academic performance. While prior research has acknowledged engagement as a key predictor of learning outcomes, this study theoretically positions cognitive engagement as a mediating construct within an AI learning framework. The findings suggest that the findings indicate that ChatGPT-assisted learning effectiveness is positively associated with academic performance both directly and indirectly through cognitive engagement. This refines theoretical models of student engagement by highlighting the role of conversational AI in stimulating higher-order cognitive involvement.

Third, the results extend the theoretical understanding of academic self-efficacy within SCT by demonstrating its mediating role in AI-enhanced learning environments. SCT emphasizes self-efficacy as a central determinant of behavior and performance; however, limited empirical work has examined how AI tools contribute to the development of students’ efficacy beliefs. The present study addresses this gap by showing that higher perceived effectiveness of ChatGPT-assisted learning is positively associated with students’ academic self-efficacy, which is also positively associated with academic performance. This finding broadens theoretical perspectives on the sources of self-efficacy, positioning AI-assisted feedback, guidance, and personalized support as influential efficacy-building mechanisms.

Fourth, by simultaneously examining cognitive engagement and academic self-efficacy as parallel mediators, the study offers a more nuanced theoretical explanation of how AI-assisted learning effectiveness is statistically associated with academic success through cognitive and motivational pathways. This dual-mediation framework enriches existing theoretical models by integrating both cognitive and motivational pathways, consistent with SCT’s emphasis on multiple interacting determinants of learning. It suggests that theories of AI-supported education should move beyond direct-effect models and incorporate complex internal processes to fully capture learning dynamics.

Finally, the study contributes to the contextualization of educational theory by applying SCT within the underexplored setting of tourism and hospitality higher education in an emerging economy. By validating theoretical relationships in Egyptian universities, the findings support the cross-cultural robustness of SCT while also extending its relevance to discipline-specific and regional contexts. This contextual contribution encourages future theoretical work to consider how AI-assisted learning mechanisms operate across diverse educational systems and fields of study.

## 7. Practical Implications

The findings of this study provide several practical insights for higher education institutions, educators, curriculum designers, and policymakers seeking to effectively integrate ChatGPT and similar AI-based tools into tourism and hospitality education. By clarifying how ChatGPT-assisted learning effectiveness is associated with academic performance through cognitive engagement and academic self-efficacy, the study offers concrete directions for maximizing the educational value of AI technologies.

First, the findings provide several practical considerations for higher education institutions, educators, curriculum designers, and policymakers interested in the responsible use of ChatGPT in tourism and hospitality education. However, because the present evidence is based on cross-sectional, self-reported data, the findings should be interpreted as associations rather than causal evidence that institutional adoption of ChatGPT will necessarily improve academic outcomes. Accordingly, any institutional integration should be accompanied by appropriate AI literacy, instructor guidance, assessment redesign, verification practices, and academic integrity policies. ChatGPT may be particularly useful as a supplementary learning resource for activities such as case analysis, language development, brainstorming, and problem solving when its use is aligned with explicit learning objectives.

Second, instructors are encouraged to design learning activities that actively promote cognitive engagement through ChatGPT. Rather than positioning AI tools as passive information providers, educators can use ChatGPT to stimulate critical thinking, scenario-based learning, reflective questioning, and interactive discussions relevant to hospitality and tourism contexts. For example, students can engage with ChatGPT to analyze service failure cases, simulate customer interactions, or explore destination management challenges. Such practices may support students’ involvement in learning tasks and are consistent with the positive associations observed in the present study.

Importantly, instructors should distinguish interaction with ChatGPT from genuine engagement with course content. Simply spending more time asking questions or receiving explanations from an AI system should not be treated as evidence of deeper learning. To convert AI interaction into meaningful cognitive engagement, instructional activities should require students to verify AI-generated claims against course materials or authoritative sources, identify weaknesses in AI responses, compare alternative explanations, justify which answer is more appropriate, and revise AI-generated material using their own reasoning. Such structured activities can position ChatGPT as a cognitive scaffold rather than a substitute for students’ intellectual effort. This approach is particularly important in tourism and hospitality education, where students must develop professional judgment and apply knowledge to complex and context-dependent service situations.

Third, the results highlight the importance of fostering academic self-efficacy through AI-assisted learning. Educators can use ChatGPT to provide timely feedback, scaffold complex tasks, and offer personalized explanations, which may help students feel more confident in their academic abilities. This is particularly valuable in tourism and hospitality education, where students often encounter applied, interdisciplinary, and practice-oriented content. When students perceive ChatGPT as a supportive learning aid rather than an evaluative tool, such practices may support students’ confidence and persistence in academic tasks.

Fourth, curriculum designers should embed AI-supported learning experiences that align with course objectives and learning outcomes. Structured integration of ChatGPT into assignments, tutorials, and independent study activities can help ensure that its use enhances engagement and self-efficacy rather than encouraging superficial learning. Training sessions or orientation workshops can also be introduced to help students develop effective AI literacy skills, enabling them to use ChatGPT strategically for learning rather than dependency.

Fifth, the study offers implications for professional development and capacity building among faculty members. Universities should invest in training programs that equip instructors with the pedagogical and technical skills needed to integrate ChatGPT effectively into their teaching practices. Emphasizing how AI tools can support student motivation, engagement, and confidence aligns with the principles of Social Cognitive Theory and encourages more intentional and theory-driven use of educational technology.

Finally, at the policy level, higher education authorities may consider developing flexible guidance for responsible AI-supported learning rather than promoting unrestricted adoption. Such guidance could address AI literacy, academic integrity, disclosure of AI use, verification of AI-generated information, assessment design, data privacy, and faculty development. The present findings provide an empirical basis for considering these issues in tourism and hospitality education, but they should not be interpreted as sufficient evidence for large-scale policy adoption on their own. Longitudinal, experimental, and institution-level evaluations are needed to determine whether structured AI integration produces sustained improvements in academic outcomes.

## 8. Limitations and Future Research

Despite its theoretical and empirical contributions, this study has certain limitations that should be acknowledged and addressed in future research. Recognizing these limitations provides opportunities to further refine understanding of ChatGPT-assisted learning and its role in higher education.

First, the study relies on data collected from tourism and hospitality students enrolled in Egyptian universities, which may limit the generalizability of the findings. Educational systems, technological infrastructure, and cultural attitudes toward AI-assisted learning vary across countries and academic disciplines. Future studies should replicate the proposed model in different national contexts, institutional settings, and fields of study to assess the robustness and cross-cultural applicability of the findings.

A further limitation concerns the measurement of academic performance. In the present study, academic performance was assessed using students’ self-reported perceptions rather than objective academic records. Although self-reported performance measures can capture students’ perceived academic capability and effectiveness in completing coursework, they may also be affected by social desirability, recall limitations, self-enhancement, or inaccurate evaluation of one’s own performance. This issue is particularly relevant in AI-assisted learning because students may perceive themselves as more capable when technological support is available without necessarily demonstrating equivalent improvement in objectively assessed academic outcomes. Future research should therefore combine self-reported measures with objective indicators such as cumulative GPA, course grades, standardized examination scores, assignment grades, or experimentally assessed learning outcomes. Such designs would provide stronger evidence concerning whether perceived improvements in academic performance correspond to measurable educational achievement.

Second, the use of a cross-sectional research design restricts the ability to draw strong causal inferences. Although the hypothesized relationships are theoretically grounded in Social Cognitive Theory and supported by mediation analysis, changes in cognitive engagement, academic self-efficacy, and performance may evolve over time. Longitudinal or experimental research designs would enable future scholars to examine how sustained exposure to ChatGPT-assisted learning influences students’ motivational and cognitive processes across academic semesters or programs.

Third, this study focuses on cognitive engagement and academic self-efficacy as mediating mechanisms, consistent with Social Cognitive Theory. However, learning in AI-supported environments is likely influenced by additional psychological and contextual factors. Future research could extend the model by examining other mediators or moderators, such as learning strategies, intrinsic motivation, academic anxiety, digital literacy, or trust in AI. Exploring moderating effects of prior AI experience or technological readiness may also yield valuable insights.

Fourth, the study treats ChatGPT-assisted learning effectiveness as a general construct without distinguishing between different types or intensities of AI use. Future studies may benefit from examining how specific applications of ChatGPT—such as feedback provision, content generation, language support, or problem-solving assistance—differentially affect engagement, self-efficacy, and performance. Experimental comparisons between guided and unguided AI use could further clarify best practices for instructional design.

Fifth, while this research is grounded in Social Cognitive Theory, future studies could integrate alternative or complementary theoretical perspectives, such as self-determination theory, cognitive load theory, or technology acceptance models. Theoretical triangulation may offer a richer explanation of how students interact with AI tools and how these interactions translate into meaningful academic outcomes.

Finally, although the questionnaire was available in both Arabic and English, the study did not directly measure participants’ English-language proficiency, prior familiarity with generative AI, attitudes toward instructor authority, or specific patterns of technology acceptance and assessment experience. These factors may influence how students perceive and use ChatGPT and, consequently, how ChatGPT-assisted learning relates to cognitive engagement and academic self-efficacy. The findings should therefore be interpreted as reflecting the particular educational and institutional environment of Egyptian public universities rather than as universally applicable relationships. Future research could explicitly measure language proficiency, generative-AI literacy, technology acceptance, instructor–student relational expectations, and assessment characteristics and examine whether these factors moderate the relationships identified in the present model. Comparative studies across countries, educational systems, and linguistic contexts would also help determine which findings are context-specific and which represent more general mechanisms of AI-assisted learning.

## Figures and Tables

**Figure 1:**
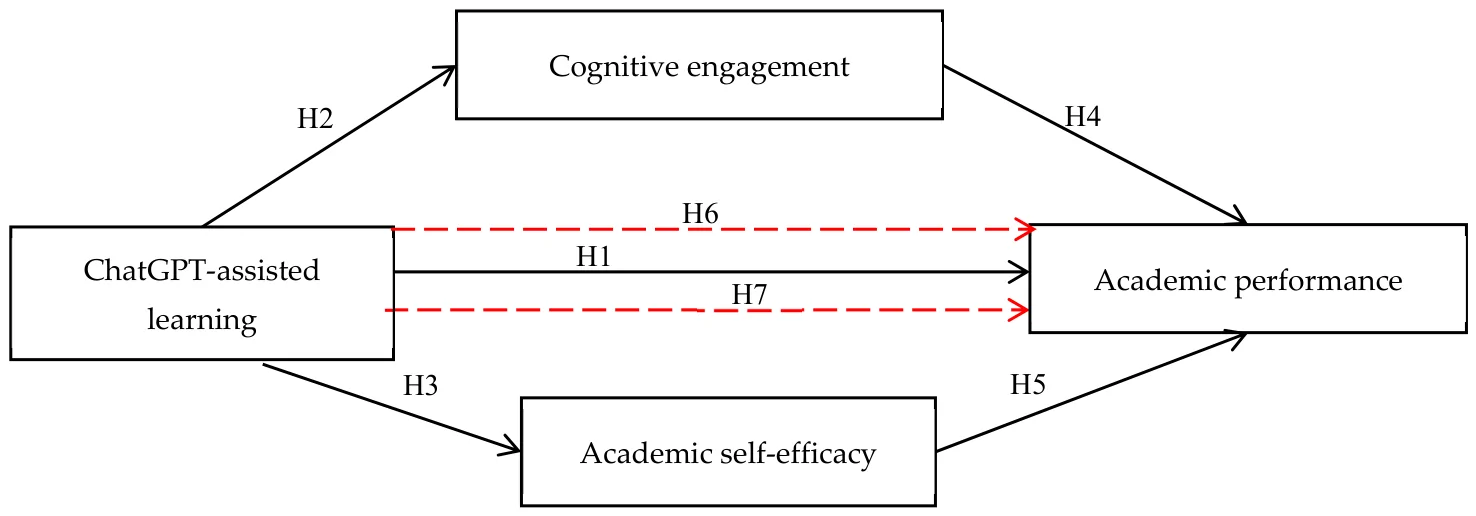
Research Conceptual Model.

**Figure 2:**
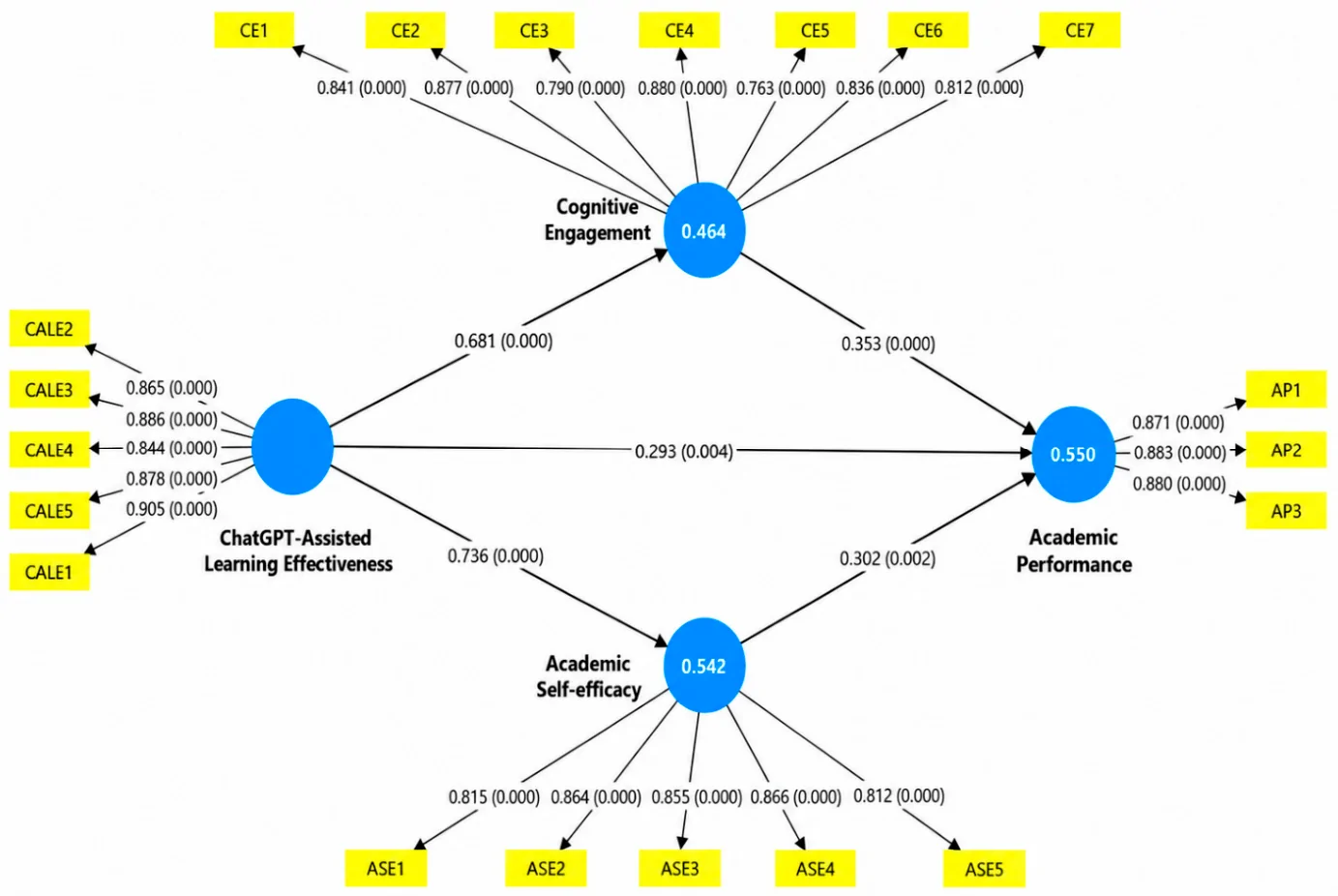
Research final model.

**Table 1:** Measurement Model Assessment.

Scale Variables	Λ	T-Value	*p*-Value	VIF
***ChatGPT-Assisted Learning Effectiveness: (α = 0.924, CR = 0.943, AVE = 0.767)***				
CALE-1	0.905	6.597	0.000	3.445
CALE-2	0.865	6.607	0.000	2.727
CALE-3	0.886	6.969	0.000	3.048
CALE-4	0.844	5.474	0.000	2.449
CALE-5	0.878	8.689	0.000	2.791
***Cognitive Engagement: (α = 0.921, CR = 0.939, AVE = 0.688)***				
CE-1	0.841	6.470	0.000	2.477
CE-2	0.877	5.689	0.000	2.449
CE-3	0.790	7.054	0.000	2.130
CE-4	0.880	5.856	0.000	2.510
CE-5	0.763	6.547	0.000	2.089
CE-6	0.836	6.152	0.000	2.624
CE-7	0.812	4.096	0.000	2.315
***Academic Self-efficacy (α = 0.898, CR = 0.925, AVE = 0.710)***				
ASE-1	0.815	3.177	0.000	2.049
ASE-2	0.864	5.759	0.000	2.600
ASE-3	0.855	8.604	0.000	2.437
ASE-4	0.866	9.207	0.000	2.687
ASE-5	0.812	6.281	0.000	2.046
***Academic Performance: (α = 0.851, CR = 0.910, AVE = 0.771)***				
AP-1	0.871	4.611	0.000	2.033
AP-2	0.883	8.665	0.000	2.136
AP-3	0.880	4.189	0.000	2.091

**Table 2:** Discriminant Validity Assessment (Fornell–Larcker Criterion and HTMT Ratio).

Construct	CALE	CE	ASE	AP
ChatGPT-Assisted Learning Effectiveness (CALE)	**0.876**			
Cognitive Engagement (CE)	0.536 [0.604]	**0.829**		
Academic Self-efficacy (ASE)	0.418 [0.572]	0.581 [0.631]	**0.843**	
Academic Performance (AP)	0.636 [0.541]	0.656 [0.651]	0.439 [0.529]	**0.878**

Note: Diagonal values in bold represent the square root of the average variance extracted (AVE). Off-diagonal values represent the correlations between constructs. Values in brackets represent the heterotrait–monotrait (HTMT) ratios.

**Table 3:** Results of Hypothesis Testing.

	B	T-Value	*p*-Values	f^2^	Interpretation
**Direct Effect**					
(H1) ChatGPT-Assisted Learning Effectiveness → Academic Performance	0.293	2.880	0.004 **	0.056	Small
(H2) ChatGPT-Assisted Learning Effectiveness → Cognitive Engagement	0.681	6.152	0.000 ***	0.366	Large
(H3) ChatGPT-Assisted Learning Effectiveness → Academic Self-efficacy	0.736	5.839	0.000 ***	0.179	Medium
(H4) Cognitive Engagement → Academic Performance	0.353	4.508	0.000 ***	0.081	Small
(H5) Academic Self-efficacy → Academic Performance	0.302	3.075	0.002 **	0.061	Small
**Indirect Effect**					
(H6) ChatGPT-Assisted Learning Effectiveness → Cognitive Engagement → Academic Performance	0.240	4.409	0.000 ***	—	
(H7) ChatGPT-Assisted Learning Effectiveness → Academic Self-efficacy → Academic Performance	0.223	3.117	0.002 **	—	
**Endogenous Construct**	**R^2^**	**Adjusted R^2^**	**Q^2^**		
Cognitive Engagement	0.464	0.463	0.438		
Academic Self-efficacy	0.542	0.541	0.517		
Academic Performance	0.550	0.549	0.487		

Note: (*p* < 0.01 = **, *p* < 0.001 = ***).

## Data Availability

Data are available upon request from researchers who meet the eligibility criteria. Kindly contact the first author privately through e-mail.

## Appendix A. Measurement Scales

ChatGPT-Assisted Learning Effectiveness (CALE) - Using ChatGPT improves the efficiency of my learning. - Using ChatGPT improves my overall learning performance. - Using ChatGPT increases my motivation to learn. - Using ChatGPT increases my confidence in my ability to learn effectively. - Using ChatGPT gives me access to additional learning resources that support my learning. Cognitive Engagement (CE) *When using ChatGPT for learning:* - I try to connect information provided by ChatGPT with other knowledge I have learned. - I try to relate different pieces of information from my course materials and ChatGPT in new ways. - I carefully examine ChatGPT’s explanations to develop a deeper understanding of the topic. - I use examples or explanations provided by ChatGPT to help me understand difficult concepts. - I analyze the information provided by ChatGPT rather than simply accepting it as correct. - I think about different ways to solve a problem or answer a question when using ChatGPT. - I apply what I learn through ChatGPT to new problems, questions, or situations. Academic Self-Efficacy (ASE) *When learning with ChatGPT:* - I am confident that I can master the skills and knowledge required in my courses. - I can successfully complete even the most difficult learning tasks when using ChatGPT. - I am confident that I can figure out how to solve difficult academic problems with the help of ChatGPT. - Even when the learning material is difficult, I am confident that I can understand and learn it using ChatGPT. - I can successfully complete most of my academic learning tasks with ChatGPT if I persist and do not give up. Academic Performance (AP) - I am confident about the adequacy of my academic skills and abilities - I feel competent in conducting my course assignments - I have learned how to perform my coursework in an efficient manner and successfully.
